# Ecology of Pathogens and Antibiotic-resistant Bacteria in Environments: Challenges and Opportunities

**DOI:** 10.1264/jsme2.ME3401rh

**Published:** 2019-03-30

**Authors:** Satoshi Ishii

**Affiliations:** 1 Department of Soil, Water, and Climate, University of Minnesota 439 Borlaug Hall, 1991 Upper Buford Circle, St. Paul, MN 55108 USA; 2 BioTechnology Institute, University of Minnesota 140 Gortner Laboratory of Biochemistry, 1479 Gortner Avenue, St. Paul, MN 55108 USA

Various bacteria can cause human diseases. They spread directly from person to person or indirectly via various environmental matrixes, such as food and water. Major food- and waterborne pathogens include *Campylobacter*, *Listeria*, *Salmonella*, *Shigella*, Shiga toxin-producing *Escherichia coli*, *Salmonella*, *Yersinia*, and *Vibrio* (31). Most of these pathogens spread through the fecal-oral route. Their primary hosts include humans, farm animals (*e.g*., cows, pigs, and chickens), and wildlife (*e.g*., deer, birds). These hosts contribute to the spread of pathogens. For example, geese and other birds are known to harbor diverse *Campylobacter* (29, 40, 59) and *Salmonella* spp. (40). However, some of these pathogens also survive for long periods of time and even grow in environments such as water, soil, sediment, and algae (13, 22, 32), in many cases in association with or by forming biofilms (35, 36, 52).

Since difficulties are associated with detecting various pathogens in a timely manner, the microbial quality of food and water has been monitored using so-called fecal indicator bacteria (FIB), such as *E. coli* and enterococci (22, 24). Although their primary habitats are the gastrointestinal tracts of warm-blooded animals (18, 49), some FIB strains are more adapted to soil or other environments (22, 24, 37). Moreover, alternative FIB, such as *Bacteroides*, have been used to identify the occurrence of pathogens and their potential sources of contamination (28, 59). However, poor correlations have been reported between pathogen and FIB concentrations (23, 58), which limits the use of FIB for predicting the occurrence of pathogens.

Some opportunistic pathogens, including *Mycobacterium avium* and *Legionella pneumophila*, are not of a fecal origin. These opportunistic pathogens use environments such as water distribution systems (11, 14, 15) and showerhead biofilms (12) as their primary habitats, and occasionally infect humans to cause diseases. Furthermore, various environmental bacteria have been reported as emerging pathogens. Among these, *Arcobacter* spp. are of great interest because this genus is frequently and abundantly detected in many wastewater treatment plants (10, 50). This genus is phylogenetically closely related to *Campylobacter*, but is metabolically more versatile and can grow at relatively low temperatures and with a wider range of O_2_ concentrations (9). Some members of *Arcobacter* have also been reported to form symbiotic relationships with protists (16). A better understanding of the ecology of these environmental pathogens is essential for preventing their occurrence and spread (39, 47).

Antibiotics are one of the most important scientific discoveries to combat pathogens. Antibiotic treatments save millions of lives each year worldwide. However, nearly a century of antibiotic use and misuse has resulted in the evolution of the resistance of bacterial pathogens to most antibiotics approved for medical use (56). In addition to clinical settings, antibiotic resistance is an issue in environments. Raw sewage is one of the major reservoirs of antibiotic-resistant bacteria (ARB) and antibiotic resistance genes (ARGs) (45). Farm animals, including cows, pigs, and horses, can also harbor various ARB, some of which are pathogenic to humans (8, 33, 55). These ARB/ARGs contaminate surrounding and downstream environments (3, 30). Wildlife can also contribute to the spread of ARB/ARGs. Migratory birds are most likely responsible for the spread of ARB/ARGs to wide areas, including remote Arctic islands (34) and Antarctica (48).

Horizontal gene transfer (HGT) plays an important role in the spread of ARGs among diverse bacteria (4, 44). Some ARGs are found on mobile genetic elements, such as insertion sequences and transposons, and can be transferred between cells when mediated by plasmids, integrative conjugative elements (ICEs), or bacteriophages (44). The HGT of ARGs can occur in environments such as wastewater treatment plants (45). In addition to conjugation (mediated by plasmids and ICEs) and transduction (mediated by bacteriophages), ARGs can be transferred between cells via extracellular vesicles (6, 53, 54) as well as when bacteria take up naked DNA (*i.e*., transformation). A recent study demonstrated that the grazing activities of Ciliates enhanced the release of ARGs from bacterial cells into the environment (5). These naked ARGs can be taken up by bacteria, thereby transforming them to be resistant to antibiotics. To prevent the spread of ARB/ARGs, it is necessary to understand the mechanisms and frequencies of ARG acquisition in environments.

The major challenges associated with the study of pathogens and ARB/ARGs in environments include (i) various types of targets, (ii) low concentrations, (iii) intra-species diversity, (iv) previously uncharacterized target genes, and (v) the occurrence of HGT. However, we can see great opportunities in these challenges. Recent technological advances have provided various tools to explore these opportunities. Three notable tools are briefly summarized below.

## High-throughput quantitative PCR

Quantitative PCR (qPCR) is commonly used to detect pathogens and ARGs. The advantages of qPCR include its high sensitivity and specificity. However, with conventional qPCR, a large number of runs are needed to detect many targets. To overcome this issue, high-throughput qPCR platform has been developed using microfluidic technology. In microfluidic qPCR (MFQPCR), multiple qPCR assays are simultaneously run for many samples in nanoliter-volume chambers that are present in high densities on a chip. MFQPCR technology has been used to quantitatively detect various pathogens and ARGs in many environmental samples (1, 7, 23, 34, 46, 48, 57). Amplicons generated on the chip can also be recovered and sequenced for further analyses (41). The MFQPCR approach is particularly useful when target pathogens/ARGs are known, their concentrations are low, and many target genes/samples need to be analyzed; therefore, it can overcome challenges (i) and (ii) described above.

## Metagenomics and amplicon sequencing

High-throughput sequencing technology has greatly advanced our understanding of microbial ecology in various environments (19, 38). Culture-independent, high-throughput sequencing of the 16S rRNA gene fragment is most commonly performed (21, 60); however, other applications, such as (meta)genomics and (meta)transcriptomics, are also frequently used (19, 25, 38). Since pathogenic and non-pathogenic strains are both present within a genus/species (*e.g*., pathogenic *E. coli*), it is difficult to identify the presence of pathogens in a sample by the high-throughput 16S rRNA gene sequencing approach alone. However, sequencing the amplicons of pathogen-specific genes is useful for analyzing the diversities of target pathogens without bacterial isolation (15, 59), and can be used to rapidly identify the sources of pathogen contamination. This approach can overcome challenge (iii) described above.

A metagenomic approach is also useful for detecting ARGs (2, 26, 27). Since this approach is independent of PCR bias, it can detect previously unknown ARGs, and therefore, overcome challenge (iv) described above. However, it could be difficult to detect genes that are present at low abundance. Furthermore, it would become expensive when analyzing many samples.

## Single-cell analysis

Various single cell-based approaches have been developed and applied to detect, quantify, isolate, and identify target pathogens and ARB. For example, a simple improvement in the fluorescence *in situ* hybridization (FISH) protocol greatly increased the detection efficiencies of *Enterobacteriaceae* cells (17). The combination of direct viable counts, multi-probe FISH, and solid-phase cytometry allows researchers to quantify viable *Vibrio* spp. (13). Fluorescently labeled *E. coli* O157:H7 cells can be individually isolated by flow cytometry-fluorescence-activated cell sorting (FACS) for downstream analyses (43).

In addition, a single-cell PCR or genomics approach can link the phylogeny and function of microbes. For example, single-cell PCR technology, called epicPCR (emulsion, paired isolation and concatenation PCR) (51), has been applied to identify ARB in wastewater samples without cultivation (20). In epicPCR, individual bacterial cells are stochastically encapsulated in polyacrylamide gel beads, in which fragments of the 16S rRNA gene and a functional gene, such as ARG, are co-amplified and fused. Amplified fused PCR products are recovered and sequenced to link functional gene sequence information with that of phylogenetic markers (51). Although there is room for further technological refinement (*e.g*., to make the size of beads even), this approach is particularly useful for the study of ARB because it has the ability to identify the host of ARGs in relatively high throughput. With this approach, it would be possible to culture-independently identify antibiotic-resistant pathogens, which is of the greatest concern for public health.

## Concluding remarks

As briefly summarized in this note, great opportunities are associated with studying the ecology of pathogens and antibiotic resistance in environments. The approaches introduced here have their own strengths and weaknesses. Although not explained in detail, the traditional culture-based method is still important, particularly when detecting resistance acquired by gene mutations (42). These methods complement each other and there is no true optimum method. Researchers need to select the most suitable approaches depending on their scientific objectives and goals.

The ecology of pathogens and antibiotic resistance in environments is one of the hot topics in environmental microbiology and microbial ecology. This topic is among the scope of *Microbes and Environments*, and its editorial board welcomes the submission of manuscripts on this topic to our journal.
